# The Diagnosis of the Internal Secretory Diseases

**Published:** 1916-12

**Authors:** Henry R. Harrower

**Affiliations:** M. D., F. R. S M. (Lond.), Los Angeles, California


### The Diagnosis of the Internal Secretory Diseases.

BY HENRY R. HARROWER, M. D., F. R. S M. (LOND.), LOS ANGELES, CALIFORNIA.

### VII.

### THE DISEASES OF THE THYMUS.

Disorders of the thymus gland are not common, though they are undoubtedly more frequent than is supposed. The growing appreciation of the important work and relations of the ductless glands, including of course the thymus, has sharpened our sensibilities and powers of observation, and in consequence thymus disease is being diagnosed more often, thus opening up still another field for therapeutic effort.

There always has seemed to be an element of mystery about this gland, due possibly to the strangeness and suddenness of deaths of thymic origin. However, this is being replaced and a number of points have been established which enable us to consider thymus disease with prospective advantage.

As with the other ductless glands, we may find a cellular enlargement of the thymus with local symptoms due to pressure; or, on the other hand, there may be change in the functional activities of the gland with varying effects upon the body as a whole. It is not yet generally conceded that the thymus is really a gland of internal secretion, although there seems to be sufficient evidence that it influences metabolism and also the work of the other ductless glands in a manner very similar to the other organs which we know are endocrine glands.

It has been suggested that the principal function of the thymus is to produce lymphocytes, and that any effects that it may exert upon metabolism, positive or negative, are due to these cells or their contents. Sajous remarks that the bulk of the available evidence points to this mode of transmission, viz., through the agency of lymphocytes which develop in the thymus. Of course, it is quite possible that these blood cells carry within themselves certain chemical substances which are very closely allied to hormones, if not actually such.

### PHYSIOLOGICAL CONSIDERATIONS.

Most authorities consider the thymus as a temporary organ which reaches its height of development about the age of two, and retrogrades slowly until puberty, at which time it is supposed to disappear, though this opinion is not unanimous. Proof that the thymus is not a lymphoid organ alone seems to be found in the clinical and experimental work which has shown an intimate relation between the action of the thymus and the metabolism of the mineral salts, especially of calcium and phosphorus. For when there is thymus disfunction the chief organs to suffer from the resultant chemical changes are the bones, muscles and, perhaps, the nerves, in the order named.

There is an abundance of evidence connecting the activities of the thymus with those of the gonads. It seems that the thymus antagonizes the action of the sex glands, and that increased thymus function, especially during the period of development, causes deficient reproductive development; while, on the other hand, deficient thymus activity may cause an increase in the growth and function of the gonads. At least, we know that if the thymus does not retrograde in the usual manner at puberty there likely may be evidences of defective sexual development.

Another important clinical fact which indicates another physiologic intimacy of the thymus is found in its relation to idiocy in children. It has been remarked that a large percentage of idiotic children have no thymus at all. Morel reports that of over four hundred idiotic children with normal thyroids coming to autopsy, over 75 per cent possessed no thymus. In passing, it is interesting to note that Klose has experimentally shown that thymectomy in dogs is followed by a gradual change in the mental powers until a condition which he terms "idiotia thymopriva" is present. While this does not necessarily prove that athymia is the cause of idiocy, it is at least a very suggestive finding and one which has been well established by many investigators.

### THYMUS INSUFFICIENCY.

Experimental proof is at hand to show that the removal of the thymus from animals causes a decided reduction of growth—dwarfism. It is not improper to presume that this holds good with children. At least there is a probable thymic element in dwarfism, and support of this is found in several communications which report benefit following thymus feeding in certain cases where weight is low and the height reduced.

Certain nutritional disorders in children, notably marasmus, are quite commonly associated with thymic atrophy, and some interesting clinical proof of this is available. Deficient children, especially when there are disturbances in bone growth and development, should always be considered as thymus cases until definitely proved not to be suffering from hypothymism.

Naturally hypothymism is not to be expected in adults, for the gland normally becomes inactive at or near puberty. However, individuals with thymus dyscrasias in childhood may retain certain chemico-nutritional disorders as a result of the previous disordered function of this gland.

The blood changes are not characteristic; but one frequently finds hypothymism accompanied by anemia and especially lymphocythemia. Reduced coagulability is also common, and frequent bleeding at the nose may be the first indication of thymus disorder. Reference has already been made to the relation of hypothymism (or athymia) to mental insufficiency. Still another incidental defect has been connected with dysthymism. Browning states that there is a relationship between the thymus gland and stammering. While all cases with an enlarged thymus do not stutter, all stutterers will be found to have an enlarged gland. This is denied by such authorities as MacCuen Smith, but is worth remembering. After all success in the detection and treatment of ductless glandular disorders is attained by noting insignificant things and the writer has "got on to" at least one case of endocrine disorder by noting that the patient stuttered.

One frequently notes a peculiar condition of hairlessness (especially of the head and face) and a yellowish, parchment-like skin in pluriglandular dyscrasias in which the thymus element is or has been prominent. Parenthetically it may be well to remark that Sajous suggests that progeria or premature senility (usually in children) is really due to thymus disease.

Some of the findings in experimental and clinical work are sometimes contradictory, and the reason for this is due to the fact that the endocrine organs are so intimately connected with one another. At one time a certain hormone seems to be in the ascendency whereas at another it is deficient. As an instance of this a case of presumed hypothymism with retarded growth and sexual development was treated with thymus substance for some months with a remarkable increase in height and general progress, though it must be recalled that theoretically the removal of the antagonism of the thymus (as in hypothymism) should favor functional gonad activity and the development and other results thereof.

### HYPERTHYMISM.

Hyperthymism is not a common or easily diagnosed condition. It is rarely found unaccompanied by other ductless glandular disorders, indeed it is a disorder which one should be ready to look for mainly in connection with certain forms of thyroid excess. A number of experiences in the literature on this subject indicate that one should carefully look for an enlarged thymus and evidences of its excessive activity in every case of Graves' disease, and particularly before surgical intervention is undertaken.

After a careful search both of the literature and numerous unpublished hospital records, Matti collated 133 cases of sudden death in hyperthyroidism in which a post-mortem examination had been held, and in 98 cases, or 74 per cent, a hyperplastic thymus was found. Such records emphasize the advice just given regarding the relation of thymus disorder with Graves' disease.

A number of deaths have followed thyroid operations, due to thymus complications. Not a few times a share, as least, of the heart and nervous symptoms attributed to hyperthyroidism has been due to a concomitant hyperthymism. In this connection, it must be emphasized that while an enlarged thymus is usual in such cases, there is no doubt that the degree of thymotoxemia may have little to do with the size of the gland.

Experimentally and clinically excessive thymus function is accompanied by severe general nervousness, tremor and a rapid irregular pulse. Thymotoxemia of this character may be amenable to roentgenization of the thymus area.

There is a somewhat rare thymus type of adiposity which is usually accompanied by lymphatic tendencies, and in which one often may find a well defined thymus area on X-ray examination. In such cases myasthenia is persistent and may disappear after suitable treatment—Roentgen or surgical.

In cases with thymus disorder one usually will find a considerable increase in the number of lymphocytes in the differential blood count, and this procedure is recommended not merely when thymus disease is suspected, but in the routine clinical diagnosis of Graves' disease.

According to Paltauf, the characteristic features of hyperthymism are as follows:

1. Hyperplasia of the various groups of lymph glands, tonsils, spleen, and, of course, the thymus itself (see status thymolymphaticus).

2. Lymphocytosis, the count being increased to 50 per cent or more (i. e., increased 100 per cent or more).

3. Cardio-aortic aplasia.

4. Maldevelopment of the genital glands and their adnexa; and

5. A pale, badly nourished skin with scanty hair and an exaggerated panniculus adiposus.

It is fair to add that one rarely finds all these in a single case.

Attention has already been called to the value of the Roentgen ray in the diagnosis of thymus disorder. An enlarged thymus occasionally may be percussed as a triangular area of dullness under the manubrium of the sternum, in some cases extending outward on either side a short distance. This area of dullness may move slightly upward on extending the neck by drawing the head well back. The base of this triangle is between the sternal ends of the clavicles, and the apex between the junctions of the sternum with the second and third ribs. Halstead has noticed that downward pressure on the sternum may produce a sense of suffocation in cases of this character, which differs considerably from the normal.

It should be recalled that there is such a condition as a substernal goiter or an intrathoracic thyroid; but this may be differentiated by the somewhat higher position of the enlargement and the fact that it moves with the trachea in the act of swallowing.

Hoxie has described a symptom complex in which an enlarged thymus is accompanied by shortness of breath and discomfort in the thorax, and extreme muscular weakness. In several cases reported the asthenia was quite the most prominent subjective finding. This is of special interest, as there seems to be clinical evidence that myasthenia gravis is in some way connected with the thymus. Tom Williams has reported a case of a man with this disease who was apparently cured by the administration of thymus substance.

### THYMUS HYPERPLASIA IN CHILDREN.

We have already referred to the symptoms of thymus enlargement and hyperactivity; but thymus hyperplasia in children deserves mention by itself. It seems to be a somewhat different clinical entity not uncommonly found in infants and children and, unfortunately, too often only at the autopsy table. Many times this hyperplasia causes no well defined symptoms and is altogether latent until sudden death, the so-called "mors thymica," is the first indication that something was wrong.

In infants, where an enlarged thymus is present, the initiation of breathing may be a prolonged and difficult matter. The cyanosis present at birth may persist and the breahing may be difficult and stridorous. In such cases the outcome is often fatal after a few hours or days.

Dyspnea in children is probably the most marked symptom of thymus hyperplasia, and its presence should always cause a careful search for other associated findings. It may vary in degree, depending upon the pressure, from an insignificant stridor worse on stretching the neck or drawing back the head, to a serious and alarming air hunger.

In such cases the general health is poor. The skin has a pasty, badly nourished appearance, not unlike that of cretinism. There may be vague respiratory symptoms due to tracheostenosis, which later may develop into a peculiar harsh and intermittent cough which is sometimes erroneously called a "tooth" cough, a "stomach" cough or, for lack of a better name (much the same as "neurasthenia") a "nervous cough." This cough occasionally may be short and dry during the day and considerably worse at night. It is possible that the cough may not be due to pressure on the air passages, but to irritation of either the recurrent laryngeal or vagus nerves, although tracheal stenosis is the most usual cause.

STATUS THYMO-LYMPHATICUS.

This disorder differs somewhat from thymus hyperplasia since it is evidently an acquired condition and is more frequently observed in older children and young adults. It is a more complex condition, the hypertrophic changes in the thymus being accompanied by a general enlargement of the bronchial, mesenteric and other lymphatic glands. According to Hart, the existence of a true status lymphaticus has not yet been proved with absolute certainty. To Hart it appears that the swelling of the lymphatic apparatus represents a tissue reaction dependent on the thymus and which may show itself also in the lymphoid components of the thymus itself.

Adenoids and enlarged tonsils are usual, hence cases with a well marked adenoid facies and other evidences of lymphatic enlargement should be studied as likely cases of status lymphaticus and the thymus should be sought for and, if possible, measured. According to Bierring and others, unexplicable deafness has been found in a number of cases.

In the past status thymo-lymphaticus commonly has been diagnosed after sudden and unexplained death. We are now better posted on the symptomatology of thymus dyscrasias, and with increasing frequency this condition is detected before extreme results show themselves and in time to perform thymectomy or, perhaps, to treat the thymus with the Roentgen ray. (This operation is not to be advised save in emergencies. Such individuals do not bear an anesthetic well—thymus death has not infrequently occurred on the operating table—and where the operation seems urgently indicated, if possible a course of radiotherapy should be tried first.)

Individuals with status thymo-lymphaticus usually are the flabby, semi-obese type, with a peculiar pasty appearance of the skin of the exposed parts. Pigmentation is occasionally seen, especially in cases of Graves' disease with thymus involvement. Incidentally the records of the pathological department of the Johns Hopkins Hospital indicate that adrenal atrophy (and presumably adrenal insufficiency) is common in cases dying from status thymo-lymphaticus. Asthenia is a usual symptom and sometimes overshadows the other subjective symptoms, and, presumably, it is of adrenal origin. Such cases often suffer from severe metabolic disorders with an intoxication which is quite probably of endocrine origin.

Quite often the development of the bones is disturbed, the growth of the extremities being stunted and a condition of softening quite similar to osteomalacia has been attributed to thymus disorder. At least derangements of the calcium metabolism are quite usual in thymus disease, and there is plenty of evidence that the thymus is concerned in the regulation of the mineral balance of the body.

The circulatory system is ineffective due to hypoplastic changes in the heart and great vessels. As a result of these organic changes resistance to disease is low, "the constitution is poor" and trivial things may produce sudden death. In young individuals the abdomen frequently assumes that type known as "pot belly," and there is an important clinical connection between thymus disorder and rickets.

### THYMIC ASTHMA.

The dyspnea of thymic origin has somewhat erroneously acquired the name "thymic asthma." This is really a form of inspiratory dyspnea due most usually to tracheo-stenosis caused by pressure by an enlarged thymus. It is only one of a series of symptoms of thymus hyperplasia and is not a distinct clinical entity, nor is it amenable to treatment different from that which is directed at the removal of the thymus or, at least, the pressure that it exerts upon the structures adjacent to it.

---

Next month: "Internal Secretory Irregularities of the Gonads."

